# Exportins can inhibit major mitotic assembly events *in vitro*: membrane fusion, nuclear pore formation, and spindle assembly

**DOI:** 10.1080/19491034.2020.1798093

**Published:** 2020-08-07

**Authors:** Matthew S. Nord, Cyril Bernis, Sarah Carmona, Dennis C. Garland, Anna Travesa, Douglass J. Forbes

**Affiliations:** Section of Cell and Developmental Biology, Division of Biological Sciences 0347, University of California-San Diego, La Jolla, CA, USA

**Keywords:** Exportins, cell cycle, ranGTP, nuclear pore, spindle, assembly, nucleoporin, importins, karyopherins, Crm1

## Abstract

Xenopus

egg extracts are a powerful *in vitro* tool for studying complex biological processes, including nuclear reconstitution, nuclear membrane and pore assembly, and spindle assembly. Extracts have been further used to demonstrate a moonlighting regulatory role for nuclear import receptors or *importins* on these cell cycle assembly events. Here we show that *exportins* can also play a role in these events. Addition of Crm1, Exportin-t, or Exportin-5 decreased nuclear pore assembly in vitro. RanQ69L-GTP, a constitutively active form of RanGTP, ameliorated inhibition. Both Crm1 and Exportin-t inhibited fusion of nuclear membranes, again counteracted by RanQ69L-GTP. In mitotic extracts, Crm1 and Exportin-t negatively impacted spindle assembly. Pulldowns from the extracts using Crm1- or Exportin-t-beads revealed nucleoporins known to be essential for both nuclear pore and spindle assembly, with RanQ69L-GTP decreasing a subset of these target interactions. This study suggests a model where exportins, like importins, can regulate major mitotic assembly events.

## Introduction

The nucleus is one of the most prominent and defining structures of the eukaryotic cell. The nuclear envelope, a double membraned structural barrier, creates a highly-regulated environment that is crucial to safeguarding and controlling the genome. Nuclear pore complexes (NPCs) regulate nucleocytoplasmic communication and transport. Each individual nuclear pore complex is a massive macromolecular structure (100–120 MDa in vertebrates [1–4], composed of ~30 different proteins, collectively known as nucleoporins or Nups, with each present in 8–32 copies per pore. There are distinct classes of Nups, including central scaffold Nups, integral membrane Nups, and FG-Nups (phenylalanine-glycine/FG repeat Nups).

Transport through the NPC for large proteins, protein complexes, and RNAs is a tightly regulated process. Molecules ranging in size from ions and nucleotides to proteins less than 20–40 kDa can passively diffuse through the nuclear pore. However, proteins larger than 20–40 kDa, as well as RNAs such as tRNAs and snRNAs, in general only traverse the NPC with the aid of a karyopherin transport receptor [5–11]. Karyopherins encompass two distinct branches: *importins* and *exportins*. Importins carry specific cargos into the nucleus, while exportins move cargos out of the nucleus.

The directionality of nuclear transport is in large part controlled by the small GTPase Ran. A steep biochemical gradient of RanGTP exists across the nuclear membrane with the concentration of RanGTP high in the nucleus and low in the cytosol [12,13]. This gradient is set up by RCC1, the GTP-exchange factor for Ran, which is an exclusively chromatin-localized protein [14-24]. RCC1 activates RanGDP by exchanging its GDP for GTP, but can do so only in the vicinity of the chromosomes. In contrast, RanGAP is bound to the cytoplasmic filaments on the outer face of the nuclear pore (and is also present in the cytosol). RanGAP acts to enhance the steep gradient of RanGTP across the nuclear membrane by ensuring that Ran hydrolyzes its bound GTP to GDP at nuclear exit [5,11,25].

Importins in the cytoplasm recognize and bind to any cargo that bears a nuclear localization signal (NLS). An importin/import cargo complex travels through the nuclear pore into the nucleus, where it first encounters RanGTP. RanGTP induces a conformational change in the Importin that causes dissociation from its NLS cargo protein, completing import. Exportins, in contrast, bind cargo containing a nuclear export signal (NES) in the case of Exportin-1 or other identifiable features such in the case of other exportins. Exportin binding to cargo can occur only inside the nucleus in interphase cells as the exportins require the simultaneous binding of RanGTP to form a ternary RanGTP/exportin/export cargo complex. This ternary complex exports through the nuclear pore, where RanGAP on the cytoplasmic filaments stimulates RanGTP to hydrolyze its GTP to GDP, causing the ternary export complex to disassemble, thus completing export [5,11,25]. Exportins, whether carrying cargo or not, have a separate binding ability to nucleoporins (Nups) that allows them to pass by sequential Nup binding through the nuclear pore.

Past studies, both *in vivo* and *in vitro*, have further shown that certain importins also have key functions in *mitosis*, where they have been shown to regulate major mitotic assembly events. The use of *in vitro* extracts derived from *Xenopus laevis* eggs has been crucial in understanding the scope of karyopherin function in mitosis. *In vivo*, in prometaphase the spindle forms around the condensed duplicated chromosomes; the chromosomes align in metaphase to be distributed evenly to the newly forming daughter cells in anaphase. Finally, in telophase the chromosome sets are each encompassed by double nuclear membranes containing nuclear pores. *In vitro* extracts of *X.laevis* eggs can be either interphase or mitotic, depending on the initial cell cycle stage of the eggs used to prepare the extracts. This duality has allowed experimenters to dissect the roles of a single protein in different cell cycle states without the constraints of working with intact cells. Indeed, mitotic extracts have proved valuable in the characterization of the events of mitotic spindle assembly. Interphase extracts have been equally invaluable in the study of molecular events, such as DNA replication, nuclear import, nuclear membrane assembly, nuclear pore assembly, nuclear size/scale [7,26–32].

Using mitotic extracts to examine spindle assembly led to the unexpected finding that an interplay between importin beta and RanGTP are key to how the cell localizes spindle assembly to occur only around the mitotic chromosomes. It was found that at mitosis importin beta binds to and inhibits a number of key spindle assembly factors throughout the mitotic cell *in vivo*, or throughout the mitotic *Xenopus* egg extract *in vitro*. However, the mitotic chromosomes, which contain bound RCC1, continue to produce RanGTP. The high concentration of RanGTP around the mitotic chromosomes frees nearby spindle assembly factors from importin beta inhibition, causing spindle assembly to occur specifically in the location of the mitotic chromosomes. Thus, RanGTP is said to act as a ‘GPS’ orchestrating where spindle assembly occurs [33–40]. Much of this pathway was worked out in *in vitro* mitotic *Xenopus* egg extracts. Further work with Importin β revealed a wide array of negatively regulated targets critical for spindle assembly [for review, see [7,36,41–45].

It was subsequently found that excess Importin β negatively also regulates other cell cycle assembly events *in vitro*, specifically the fusion of nuclear membranes and, separately, the assembly of nuclear pores within the nuclear membranes [45–49]. Here again inhibition by importin β is counteracted near chromatin where RanGTP is produced, allowing targeted nuclear assembly to occur specifically around the mitotic chromosomes. Transportin, a second importin, was also shown to be able to regulate spindle, nuclear membrane, and nuclear pore assembly, in a regulatory pathway parallel to that of Importin β; Transportin does not titrate RanGTP, but binds to specific targets [50,51]. In the case of nuclear pore assembly by these two importins, their targets include certain dual function nucleoporins that are localized to the nuclear pore in interphase and the mitotic kinetochore in mitosis [41,50]. The data supports a model where the two importins bind to and inhibit pore assembly factors in areas distant from mitotic chromosomes but release them near mitotic chromosomes in response to RanGTP produced by chromatin-bound RCC1. Indeed, one of the karyopherin-inhibited targets, for example, was revealed in importin beta- and transportin-pulldowns to be the DNA-targeting protein and dual NPC/kinetochore protein, ELYS [50,52,53]. In sum, it appears that RanGTP acts as a molecular ‘GPS’ to establish a spatial gradient around the chromosomes that denote not only where the mitotic spindle should form early in mitosis (prometaphase), but also where the nuclear membranes and NPCs should form later in mitosis (telophase) [36,41].

Exportins have yet to be studied *in vitro* for an effect on mitotic assembly events. The exportins have a distinct and interesting set of cargoes: Crm1 (Exportin-1, Xpo1) is the major export receptor for nuclear proteins that exit the nucleus; Crm1 recognizes nuclear proteins that possess a leucine-rich NES [54–60]. Of all the exportins, Crm1 has the largest and most diverse set of cargo, ranging from single proteins such as p53 to snRNPs to full ribosomal subunits [61–64]. Interestingly, in certain cancer types overexpression of Crm1 leads to the excessive nuclear export and subsequent inaccurate cytoplasmic localization of anti-tumor proteins and pro-apoptosis proteins. Indeed, the mislocalization of p53, BRCA1 and other cell cycle regulators to the cytoplasm directly contributes to the proliferation of these cancer cell types. This has led to the development of new targeted inhibitors of Crm1, known as selective inhibitors of nuclear export or SINEs, and their increasing use as a novel and productive chemotherapy in the clinic [65–67].

Here we have used *Xenopus* egg interphase extracts to address whether exportins play a role in nuclear membrane fusion and nuclear pore formation in newly forming nuclei. We also used mitotic extracts to preliminarily explore a role for Crm1 and Exportin-t in spindle assembly. The exportins tested include Exportin1/Crm1, which exports NES-bearing protein cargos, Exportin-t, which exports tRNAs, and Exportin-5, which exports pre-miRNAs, double stranded RNA-binding proteins and other cargos [8,68,69] [59,70,71]. Our results reveal that the exportins tested here indeed have inhibitory effects on multiple mitotic assembly events.

## Materials and methods

### Expression and purification of recombinant proteins and antibodies

Recombinant proteins including: GST, GST-hTransportin, GST-xCrm1, GST-hExportin-t, 6xHis-hExportin-5, and GST-RanQ69L were expressed in BL21 (DE3) cells (New England BioLabs, C25271) by growing 1 L at 37⁰C for 3 hours or until the OD_600_ was ~0.500. Protein expression was induced by adding IPTG (0.5 mL of 1 M stock) to a final concentration of 0.5 M and growing cells overnight at 16⁰C. The cells were collected by centrifugation, resuspended in 25 mL of bacterial lysis buffer (300 mM NaCl, 50 mM Tris, pH = 7.5) plus 5 mg lysozyme (BioPioneer, C0021) and frozen at −80⁰C. Cells were thawed on ice, then sonicated and centrifuged in an SS-34 rotor (Sorvall) to clear the lysate of insoluble material (24,000 G, 45 minutes, 4⁰C). The lysate was applied to Glutathione Agarose 4B beads (Prometheus Protein Biology Products, 20–542) or Super Ni-NTA Agarose Nickel beads (Lambda Biotech, G202) and the expressed proteins purified according to manufacturers’ instructions. Proteins were stored in aliquots at −80⁰C in Egg Lysis Buffer [ELB; 250 mM Sucrose, 2.5 mM MgCl_2_, 50 mM KCl, 10 mM HEPES, and the pH was adjusted to 7.8 with KOH [47]]. Antibodies used in this study were generated from rabbit serum against *Xenopus* anti-Nup98 [51] and *Xenopus* anti-Nup133 [47,50].

### Spindle assembly assays

Spindle assembly assays were adapted from previously described protocols [50,51]. In brief, *Xenopus* eggs were collected and were lysed in XB+EGTA (50 mM sucrose, 1 mM MgCl_2_, 100 mM KCl, 10 mM HEPES, 7 mM EGTA, pH = 7.7–7.8 with KOH) by centrifugation in a TOMY TX-160 centrifuge at 15 K RPM for 20 minutes at 4⁰C. The crude cytosol was removed and supplemented to 50 μg/mL Cytochalasin B (EMD Millipore Corp, 250233–5 MG), 10 μg/mL Aprotinin and 10 μg/mL Leupeptin (USB, 11388 and 18413, respectively). The mitotic cytosol was then centrifuged, as described above, and the supernatant was removed and kept on ice until ready to use. For each spindle assembly reaction, 20 μL of mitotic extract, 1.6 μg of Rhodamine-labeled tubulin (Cytoskeleton, TL590 M-A) and 1.5 μL ATP regenerative (200 mM phosphocreatine, 1.6 mg/mL creatine kinase, 20 mM ATP, 20 mM MgCl_2_, 2 mM EGTA) mix was added to each 1.5 mL tube. The recombinant protein was then added to the appropriate reaction. For the Crm1 spindle assembly experiment, recombinant proteins were added to the following final concentrations: 15 μM GST, 15 μM Transportin, and 1, 2, 8, 10, 15, and 20 μM Crm1 (Figure 3). For the Exportin-t analysis in spindle assembly, recombinant proteins were added to the following final concentrations: 15 μM GST, 15 μM Transportin, 15 μM RanGTP-Q69 L, 15 μM Exportin-t, and 15 μM RanGTP-Q69 L plus 15 μM Exportin-t. Lastly, sperm chromatin was added to a final concentration of ~3000/μL. (We refer to the DNA used to initiate mitotic spindle or nuclear assembly as sperm chromatin packets. Each packet contains the highly condensed chromosomes of a single sperm cell from a male frog.) Each reaction was allowed to progress for 1 hour at room temperature, then 2.5 μL of reaction was plated and mixed with 1 μL spindle fixation buffer (48% glycerol, 11% formaldehyde, 10 mM HEPES, pH = 7.5, 5 μg/mL Hoechst). All samples were visualized with an Axioskope 2 microscope with a 63x objective (Carl Zeiss MicroImaging).

### Membrane fusion assays

*Xenopus* high speed mitotic or interphase cytosol and membranes were prepared as previously described [47,50,51]. The membrane fusion assays in Figure 2 were conducted as previously described [50] and were analyzed on an Zeiss Axio Observer microscope with a 63 × 1.4 NA objective. Images were captured with an Axiocam 506 monochrome digital camera and analyzed using Zen 2.3 (all from Carl Zeiss MicroImaging). Additionally, images were visualized using an Axioskop 2 microscope and a 63x objective (Carl Zeiss Microimaging). Membranes were visualized with 3,3ʹ-Dihexyloxacarbocyanine iodide (DHCC; Sigma-Aldrich, 318426). Nuclear membrane exclusion capabilities were assessed by addition of 70 kDa Rhodamine-labeled Dextran (Molecular Probes Life Technologies, D1819). DNA was stained with bis-Benzimide H 33258 dye (Hoechst; Sigma-Aldrich, B1155). The reactions were set up as follows in a 1.5 mL tube: 20 μL high speed *Xenopus* interphase egg extract, 1 μL 20x clarified *Xenopus* egg membranes (prepared as above), 1.25 μL of ATP regenerative mix, 1 μL of ~50,000 sperm chromatin (~2,000 *Xenopus* sperm chromatin packets/μL final concentration), and added recombinant protein. The following recombinant proteins (final concentrations) were used in control reactions: 25 μM GST, 25 μM GST-hTransportin, 37.5 μM GST-RanQ69L-GTP, and 25 μM GST-hTransportin plus 37.5 μM GST-RanQ69L-GTP. The experimental exportin proteins added to separate reactions were as follows: 25 μM GST-xCrm1/exportin1 or 25 μM GST-hExportin-t, plus or minus 37.5 μM GST-RanQ69L-GTP. All the recombinant proteins were diluted to 200 μM, then 3.3 μL of each protein was added at t = 0 to the appropriate reaction tube, which contained 23.25 μL before the addition of the recombinant protein. The reactions were allowed to progress for 90 minutes at room temperature, which was roughly 22⁰C. For the membrane fusion assays, after 90 minutes 1 μL (2.5 μg) of 70 kDa Rhodamine-labeled Dextran was added to each reaction and then incubated on ice for 10 minutes. The reactions were then fixed by adding 9 μL of 16% paraformaldehyde (Sigma-Aldrich, P6148-500 G) in PBS (137 mM NaCl, 2.7 mM KCl, 10 mM Na_2_HPO_4_, 1.8 mM KH_2_PO_4_) to a final concentration of 4% paraformaldehyde and left on ice until microscopic analysis.

**Figure 1.**
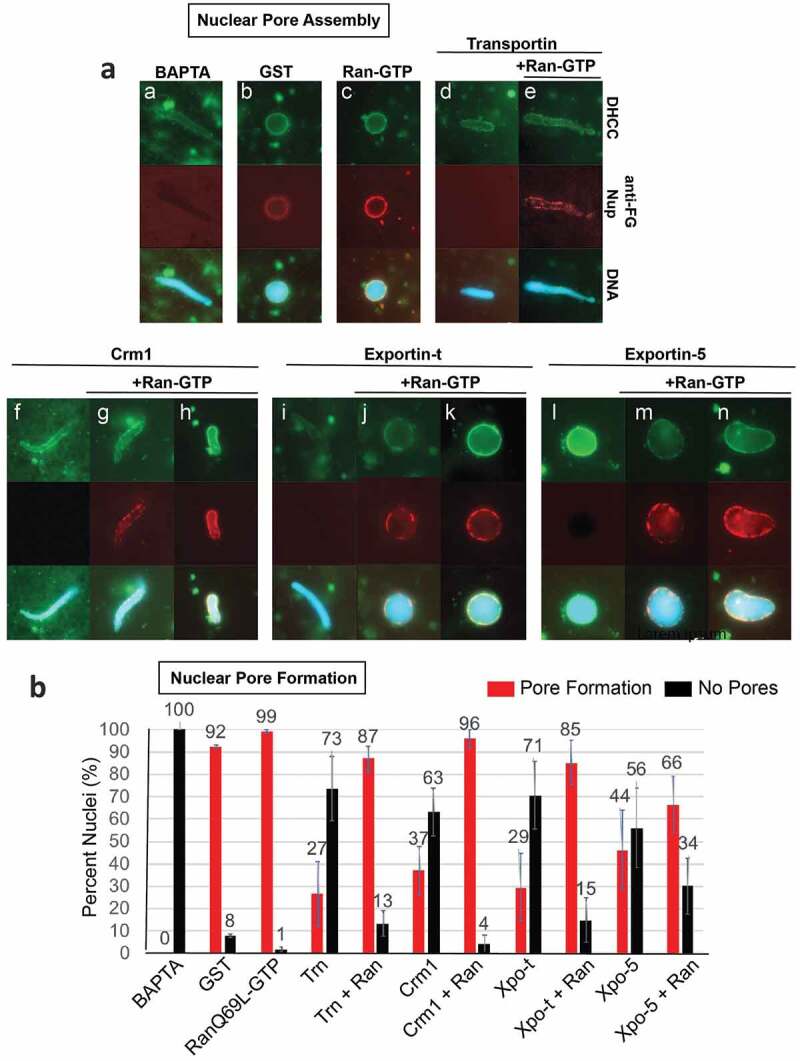
Crm1, Exportin-t, and Exportin-5 inhibit nuclear pore formation in pore-free BAPTA nuclei.

(a) BAPTA nuclei were assembled for 1 hour at room temperature, then, upon checking that nuclear pores were indeed inhibited by staining with 414, diluted 1:10 into fresh cytosol to allow release from BAPTA inhibition. In advance of diluting the BAPTA nuclei into fresh cytosol, the following was added to cytosol; (**a**: 7.5 mM BAPTA. **b**: 25 μM GST. **c**: 37.5 μM RanQ69L-GTP. **d**: 25 μM Transportin. **e**: 25 μM Transportin+37.5 μM RanQ69L-GTP. **f**: 25 μM Crm1. **g**: 25 μM Crm1 + 37.5 μM RanQ69L-GTP (partial rim). **h**: 25 μM Crm1 + 37.5 μM RanQ69L-GTP (full rim). **i**: 25 μM Exportin-t. **j**: 25μ M Exportin-t + 37.5 μM RanQ69L-GTP (partial rim). **k**: 25 μM Exportin-t + 37.5 μM RanQ69L-GTP (full rim). **l**: 25 μM Exportin-5, **m**: 25 μM Exportin-t + 37.5 μM RanQ69L-GTP (partial rim). **n**: 25 μM Exportin-t + 37.5 μM RanQ69L-GTP (full rim). Red is 414 anti-FG Nups, green is DHCC, blue is Hoechst. (b) Quantification of nuclear pore formation assays. The percentages were determined by counting at least 50 nuclei from each condition. Each experiment was repeated three times. Error bars represent the Standard Error from the Mean. Abbreviations: Trn = Transportin, Ran = RanQ69L-GTP, Xpo-t = Exportin-t, Xpo-5 = Exportin-5.

**Figure 2.**
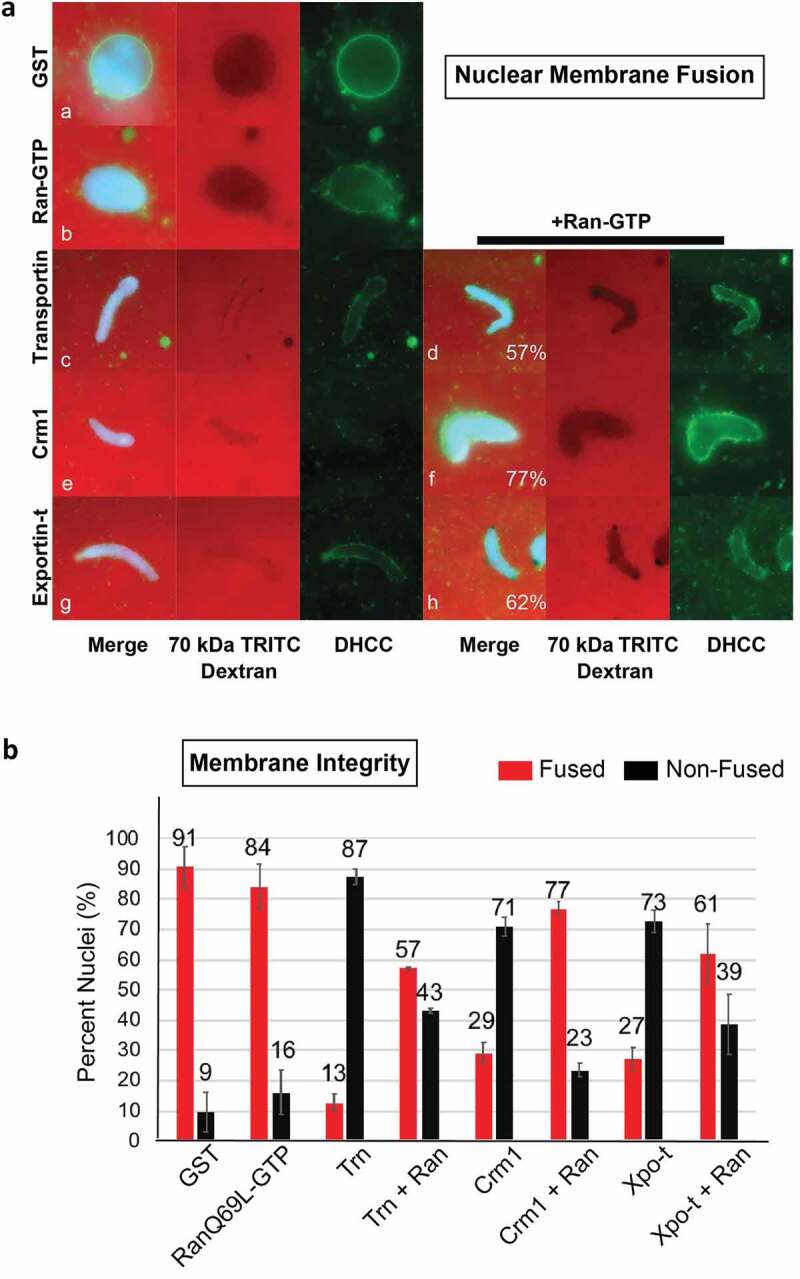
The Exportins Crm1 and Exportin-t inhibit nuclear membrane fusion and can be counteracted by RanQ69L-GTP. (a) Crm1 and Exportin-t were added to extracts together with sperm chromatin interphase egg extracts and nuclear membrane fusion was assayed after 1 hour at room temperature. The primary measure of fully fused nuclear membrane was the ability to exclude TRITC-70 kDa Dextran (red); fusion is also supported by the appearance of a smooth membrane around the DNA (green, DHCC). The conditions assayed included the following addition of recombinant protein. **a**: 25 μM GST, **b**: 37.5 μM RanQ69L-GTP, **c**: 25 μM Transportin, **d**: 25 μM Transportin + 37.5 μM RanQ69L-GTP, **e**: 25 μM Crm1, **f**: 25 μM Crm1 + 37.5 μM RanQ69L-GTP, **g**: 25 μM Exportin-t, and **h**: 25 μM Exportin-t + 37.5 μM RanQ69L-GTP. Note: RanGTP alone causes abundant membrane fusion, producing a nuclear membrane with many outfoldings, which is less smooth than seen in GST alone nuclei. (b) Quantification of membrane fusion assays. The percentages shown were determined by counting at least 50 nuclei from each condition. Each experiment was repeated three times. Error bars represent the Standard Error from the Mean. Trn = Transportin, Ran = RanQ69L-GTP, Xpo-t = Exportin-t.

**Figure 3.**
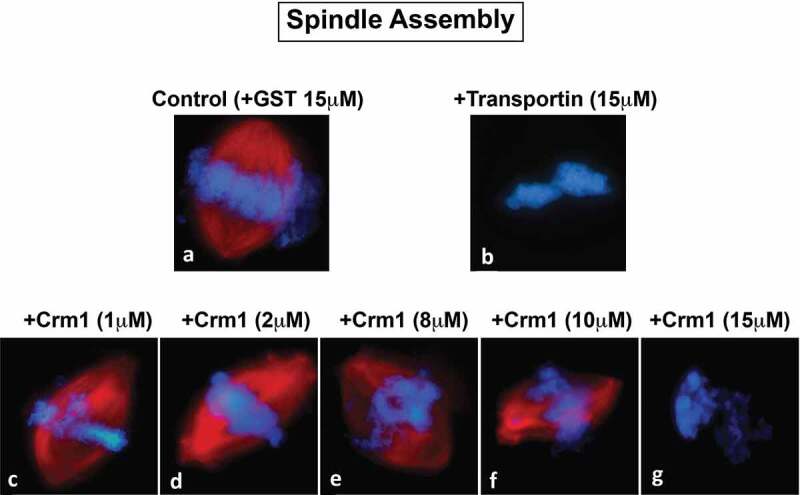
Crm1 inhibits spindle assembly. Recombinant Crm1 (c-g) was added to mitotic extract supplemented with rhodamine-labeled tubulin and compared to GST (a) and Transportin controls (b). Typical images for each reaction are shown. GST (A) addition did not interfere with the production of strong bipolar spindles, while 15 μM Transportin (B) strongly inhibited bipolar spindle formation. Low concentrations (1–2 μM) of Crm1 (c and d) had little effect on bipolar spindle formation. However, increasing concentrations (8–10 μM) Crm1 had an increasingly deleterious effect on bipolar spindle formation (e and f). Bipolar spindle assembly was completely inhibited by 15 μM Crm1 (g).

**Figure 4.**
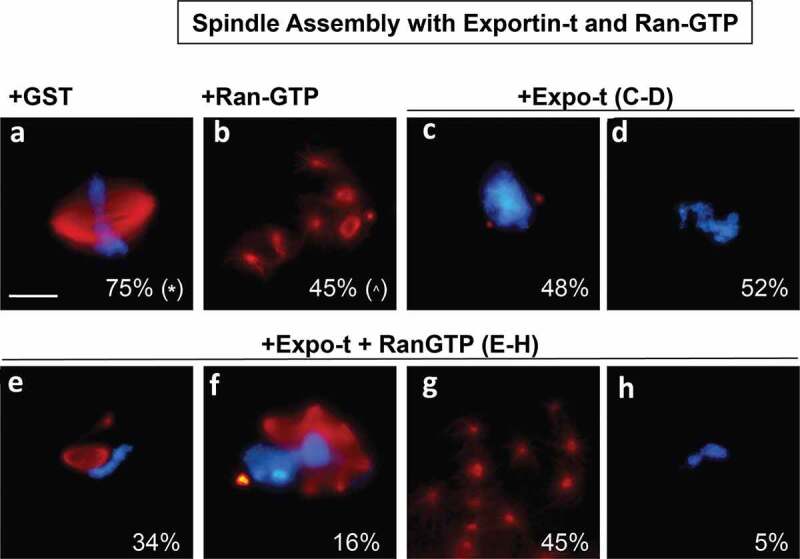
Spindle assembly with Exportin-t and RanGTP. Exportin-t was analyzed in mitotic extract to assess its effect on spindle assembly. (a): In control 15 μM GST conditions, 75% of the structures observed were bipolar spindles (* the remaining 25% of the structures were half spindles). (b): RanQ69L-GTP addition created an abundance of microtubule nucleations called asters (^ the remaining 55% of structures were bipolar spindles and multi-polar spindles). When 15 μM Exportin-t was added, two major phenotypes were observed, as shown in (c): 48% of the structures were very small microtubule nucleations on opposite sides of the mitotic chromatin, and (d): 52% were mitotic chromatin completely inhibited for spindle assembly. When 15 μM Exportin-t and 15 μM RanQ69L-GTP were added simultaneously, several phenotypes were observed. These percentage of structures observed were as follows: (e): a small spindle directly adjacent to mitotic chromatin (35%), (f): multipolar spindles around mitotic chromatin (16%), (g): groups of microtubule nucleations called asters, commonly seen in extracts with high RanGTP (45%), and (h): mitotic chromatin with no spindle associated (5%).

**Figure 5.**
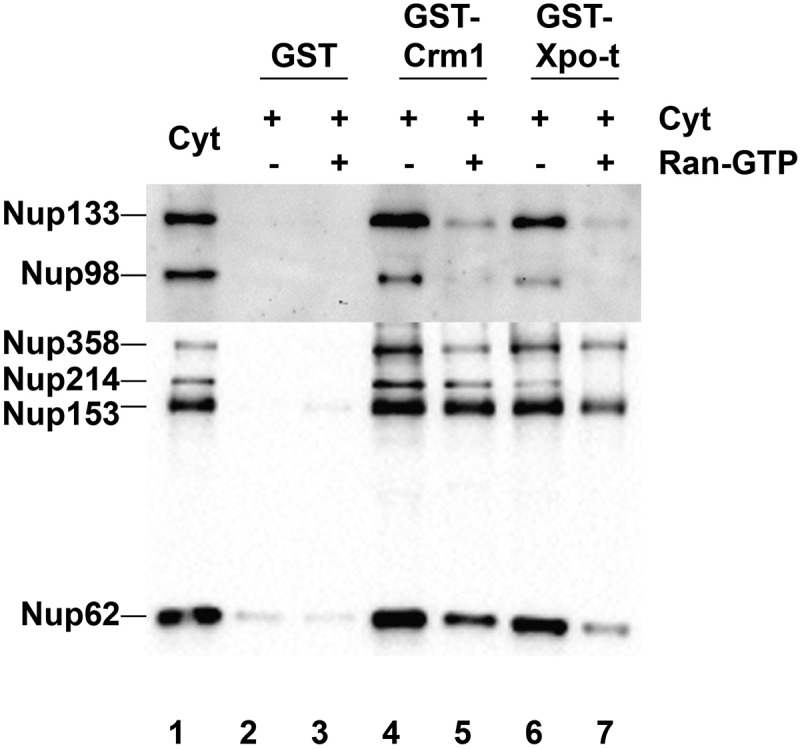
Crm1 and Exportin-t nucleoporin interactions in Xenopus egg cytosol. GST, GST-Crm1 and GST-Exportin-t were bound to glutathione beads and incubated in cytosol with or without RanQ69L-GTP. The beads were washed in PBS+0.2% NP-40. The bound proteins were eluted from the beads, resolved on a gel (lanes 2–7), then transferred and probed with the following antibodies: 414 anti-FG Nups, anti-Nup133, anti-Nup98, and anti-actin. Actin showed a clean signal for the input extract cytosol, but was not present in the GST-, GST-Crm1, or exportin-t bead pulldown lanes (data not shown). Lane 1: cytosol input (0.5 μL of interphase cytosol). Lane 2: GST. Lane 3: GST + RanQ69L-GTP. Lane 4: GST-Crm1. Lane 5: GST-Crm1 + RanQ69L-GTP. Lane 6: GST-Exportin-t. Lane 7: GST-Exportin-t + RanQ69L-GTP.

**Figure 6.**
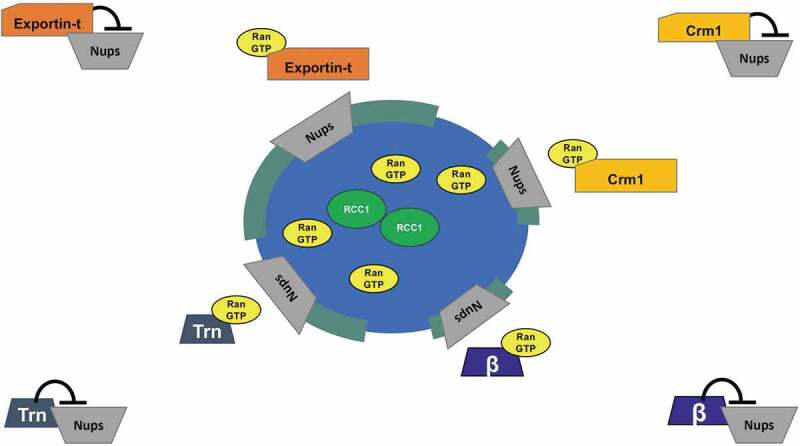
A model for Exportin action in nuclear pore assembly.

The exportins Crm1 and Exportin-t are proposed to regulate nuclear pore assembly *in vitro* by binding to critical Nup subunits in regions distant from the chromatin and preventing the subunits from assembling in those distant loci. In the model this inhibition is released around chromatin in response to the locally high concentration of RanGTP that is produced by the chromatin-bound Ran-GEF, RCC1. Because nuclear membrane assembly has not yet sealed off the genome in these early stages of nuclear assembly, RanGTP produced by RCC1 is able to locally leave the forming nucleus and release nearby soluble Nup subunits from exportin inhibition. This frees the Nup subunits to assemble into complete nuclear pore complexes in the forming nuclear membranes (green). A similar model has been proposed for the importins Importin β and Transportin (Trn) in their regulation of nuclear pore assembly during the cell cycle [41,47]. Exportin-5 (not shown) could act similarly to Crm1 and Exportin-t in the mechanism shown here.

### Nuclear pore assembly assays

Nuclear pore assembly assays were conducted by first creating pore-free nuclear intermediates termed ‘BAPTA’ nuclei (Figure 4.3), as previously described [47,50,51]. To generate BAPTA nuclei, reactions were set up in a 1.5 mL tube, as follows: 20 μL high speed interphase extract, 1 μL 20x clarified membranes, 1.25 μL of ATP regenerative mix (200 mM phosphocreatine, 1.6 mg/mL creatine kinase, 20 mM ATP, 20 mM MgCl_2_, 2 mM EGTA), ~1 μL containing 2000 sperm chromatin packets/μL, and 0.7 μL of 250 mM BAPTA in water (1,2-bis(*o*-aminophenoxy)ethane-*N,N,N′,N′*-tetraacetic acid; Calbiochem, 196419) to give a 7.5 mM final concentration of BAPTA in the reaction, and allowed to progress for 1 hour at room temperature. To ensure nuclear pores had not formed in the BAPTA nuclei, FG Nups were probed for by adding 0.5 μL of Alexa Fluor 594 anti-Nuclear Pore Complex Proteins antibody (mAb414; anti-FG Nups; BioLegend, 682202) to a 5 μL sample of each BAPTA nuclei reaction. After ascertaining the BAPTA nuclei intermediates were in fact pore-free, the BAPTA nuclei were then diluted 1:10 into fresh interphase cytosol (5 μL of BAPTA nuclei in 45 μL of fresh cytosol and 2.5 μL ATP regenerative mix). Before addition of the BAPTA nuclei, the following reactions (final concentration) were prepared in fresh cytosol as controls: 7.5 mM BAPTA, 25 μM GST, 25 μM GST-hTransportin, 37.5 μM GST-RanQ69L-GTP, and 25 μM GST-hTransportin plus 37.5 μM GST-RanQ69L-GTP. The exportin experimental conditions were as follows: 25 μM GST-xCrm1 plus or minus 37.5 μM GST-RanQ69L-GTP, 25 μM GST-hExportin-t, plus or minus 37.5 μM GST-RanQ69L-GTP, and 25 μM 6x-His-hExportin-5 plus or minus 37.5 μM GST-RanQ69L-GTP. All proteins were diluted to 423 μM in ELB, then 2.68 μL were added to the appropriate tube. One hour after dilution into fresh cytosol, 1 μL of the Alexa Fluor 594 anti-Nuclear Pore Complex Proteins antibody (mAb414; anti-FG Nups) was added to each reaction and incubated for 1 hour at room temperature. Then, 15 μL of 16% paraformaldehyde in PBS was added to a final concentration of 4%, and the reactions were left on ice until imaged. For all experiments, a minimum of 50 nuclei were counted per reaction, and each experiment was repeated at least three times. Error bars are standard error from the mean.

### GST pulldowns and immunoblots

To prepare beads for pulldowns, 180 μL of Glutathione Agarose 4B beads (Prometheus Protein Biology Products, 20–542) were washed two times with PBS, and blocked with 2.5 μL of 20 mg/mL BSA for 1 hour at room temperature. At that point, 100 μg of GST (7 μL), GST-xCrm1 (3.3 μL), or GST-hExportin-t (3.3 μL) were bound to the beads for 1 hour at room temperature while being gently mixed in a final volume of 0.5 mL of PBS. The tubes with GST-, Crm1-, and Exportin-t-bound beads were each split evenly into two 1.5 mL tubes (yielding 50 μg of bound protein per tube) and 25 μL of interphase *Xenopus* egg extract (precleared of residual membranes by centrifugation in a TOMY TX-160 centrifuge at 15 K RPM for 20 minutes at 4⁰C) was added to each tube, and the volume was brought up to 500 μL with PBS. In half of the tubes, 100 μg of 6xHis-RanQ69L-GTP (3.8 μL) was added so that each condition (GST, GST-xCrm1, and GST-hExportin-t, respectively) was also tested with added RanGTP. The tubes were mixed gently at 4⁰ for 2 hours. The tubes containing beads were then centrifuged at 3 K RPM in an Eppendorf Microcentrifuge 5415 C, and the supernatant carefully aspirated to avoid removing beads. The beads were washed three times with PBS+0.2% NP-40, then three times with PBS. The PBS was removed and the samples were resuspended and boiled at 95⁰C for 5 minutes in 30 μL of 2X gel sample buffer (31.5 mM Tris-HCl, pH = 6.8, 10% glycerol, 1% SDS, 0.005% Bromophenol Blue) and run on a 10% SDS-PAGE gel until resolved. The proteins were transferred overnight in Transfer Buffer (3 g Tris Base, 14.4 g glycine, 1 g SDS and 20% ethanol per liter) onto a nitrocellulose membrane at 30 V. The membrane was blocked in 5% milk (Apex BioResearch Products, 20–241) in TBS+0.1% Tween 20 for 1 hour at room temperature, then cut into sections for probing. Primary antibodies were prepared in the following dilutions in 5% milk in TBS+0.1% Tween 20, with 414 anti-FG antibody 1:1000, or rabbit anti-Nup133 1:200, or rabbit anti-Nup98 1:1000. The membranes were incubated overnight with the added antibodies at 4⁰, washed 3 times for 5 minutes at room temp in TBS+0.1% Tween 20. Then, HRP-conjugated Protein A for the rabbit antibodies (Invitrogen, 101023) or HRP-conjugated goat anti-mouse for the 414 anti-FG antibody (Invitrogen, 626520) were bound 1 hour at room temperature at 1:10,000 in 5% milk in TBS+0.1% Tween 20. After 3 washes, the membrane was treated with ECL Western Lighting Plus (Perkin Elmer, NEL103001EA), imaged on a ChemiDoc XRS Imaging System, and analyzed using Image Lab 4.1 (BioRad).

## Results

### Crm1, Exportin-t, and Exportin-5 addition inhibits nuclear pore assembly and RanQ69L-GTP counteracts inhibition

To investigate whether exportins can regulate nuclear envelope fusion, we used an *in vitro* nuclear assembly assay derived from *Xenopus* interphase egg extract. Such extracts are capable of assembling nuclei within 60–90 minutes. We first asked whether three specific exportins were able to inhibit nuclear pore formation *in vitro*. In this instance a well-known technique that allows separation of nuclear membrane assembly from nuclear pore assembly was used [29,47,50,51], so that nuclear pore assembly could be monitored separately. Specifically, nuclear reconstitution reactions performed in the presence of the Ca^2+^ chelator BAPTA are known to form distinct nuclear intermediates that contain smooth and intact double nuclear membranes, but are devoid of nuclear pores, termed ‘BAPTA nuclei’ or ‘pore-free nuclei’ [29]. Pore-free nuclear intermediates were produced here by nuclear assembly in 7.5 mM BAPTA, and nuclear membrane assembly was confirmed at 60‘ using the membrane dye DHCC. It is known that when such pore-free intermediates are then diluted into fresh interphase egg extract lacking BAPTA, nuclear pore assembly readily occurs by 60ʹ as observed by the presence of nuclear pore staining with fluorescently tagged anti-FG Nup antibodies [29]. Such pore-free nuclear intermediates have been used in prior studies to assess the influence during pore assembly of addition of: (a) small molecule inhibitors such as GTPγS or lipids, (b) anti-Nup antibodies, or (c) regulatory factors such as importin β or Transportin, for their effect on nuclear pore assembly [29,47,50,51,72]. In sum, BAPTA nuclei provide pore-free intermediates that allow for the specific analysis of nuclear pore formation separate from membrane fusion.

Using preformed BAPTA pore-free nuclear intermediates, which clearly lacked pore staining (Figure 1a, panel a), the effects of Crm1, Exportin-t, or Exportin-5, added with fresh BAPTA-free cytosol, on nuclear pore assembly was individually analyzed. Pore assembly was assessed using a red fluorescent antibody to FG nucleoporins (Figure 1). When GST as a positive control was added with the fresh BAPTA-free cytosol, nuclear pore assembly readily occurred by 60ʹ (Figure 1a, panel b, red). Similarly, RanQ69L-GTP and fresh cytosol promoted abundant nuclear pore assembly (panel c, red). When Transportin, previously shown to be an inhibitor of nuclear pore assembly, was added to pore-free nuclear intermediates at the same time as fresh BAPTA-free cytosol, no nuclear pore assembly was observed (panel d, red). This inhibition by Transportin was prevented if RanQ69L-GTP was included (panel e, red). RanQ69L-GTP has previously been shown to counteract the effect of Transportin on nuclear pore assembly [50,51].

The addition of the exportin Crm1 to pore-free nuclear intermediates at the same time as fresh BAPTA-free cytosol caused strong inhibition of nuclear pore formation (panel f, red channel). However, if RanQ69L-GTP was added with the Crm1 and fresh cytosol, nuclear pore formation was significantly rescued (panel g-h, red), with 90% of the nuclei formed having a red rim indicative of abundant nuclear pores.

Exportin-t (Figure 1(a), panel i, red) or Exportin-5 (panel l, red) addition to the pore-free nuclear intermediates at the time as fresh BAPTA-free cytosol also prevented visible nuclear pore formation. RanGTP inclusion with Exportin-t (panels j-k) or Exportin-5 (panels m-n) prevented such inhibition; nuclear pore complex restoration by RanGTP ranged from partial to complete restoration around the entire nuclear rim. Quantification of these results is shown in Figure 1(b), comparing the percent of total nuclei containing nuclear pores (red bars) versus the percent of nuclei which lacked nuclear pores (black bars). We conclude that the exportins Crm1, Exportin-t, and Exportin-5, when added in excess, inhibit nuclear pore assembly and that this inhibition can be counteracted by RanGTP.

### Crm1 and Exportin-t inhibit nuclear membrane fusion while RanQ69L-GTP counteracts their inhibitory effects

To analyze the potential effect of exportins on nuclear membrane assembly, exogenous Crm1 or Exportin-t was added to interphase *Xenopus* egg extract containing sperm chromatin and membrane vesicles at t = 0ʹ and the reaction was allowed to incubate for 1 hour. The fusion of vesicles to form nuclear membranes was determined by monitoring two different aspects of the reconstituting nuclei (Figure 2(a)). First and most importantly, the *integrity* of the nuclear membranes was analyzed by the ability to exclude TRITC-labeled 70 kDa dextran. Second, the *continuity* of the membrane was assessed by addition of the fluorescent membrane dye DHCC. DHCC stains membranes such as those surrounding the reconstituting nuclei, allowing for a general visualization of smooth contiguous membranes (for example, Figure 2(a), panel a) or, alternatively, bound membrane vesicles blocked in an unfused (single vesicles) or partially unfused state (runs of smooth nuclear membrane separated by regions of unfused vesicles). TRITC-70 kDa dextran exclusion is the strongest indicator of a complete nuclear membrane having formed.

In positive controls, nuclear assembly reactions with added recombinant GST produced nuclei with primarily intact nuclear membranes at t = 60ʹ, as seen both by TRITC 70 kDa dextran exclusion (Figure 2(a), panel a, red) and by strong continuous DHCC membrane staining (Figure 2(a), panel a, green). RanGTP added alone also produced intact nuclear membranes as measured by TRITC 70 kDa dextran exclusion (Figure 2(a), panel b, red); DHCC stain showed nuclear membranes with multiple oupocketings (panel b, green), as expected, as RanGTP addition is known to cause an excess of nuclear membrane fusion [47]. In a negative control, the previously demonstrated inhibitor of nuclear membrane fusion, Transportin, which is a RanGTP-sensitive import receptor [50,51], was added to a nuclear assembly reaction at t = 0. As expected, Transportin prevented nuclear exclusion of 70 kDa dextran (panel c, red) and blocked the appearance of smooth nuclear membranes (panel c, green). Notably, we found that the addition of either Crm1 or Exportin-t to nuclear assembly reactions at t = 0ʹ resulted in no exclusion of TRITC 70 kDa dextran (Crm1, panel e, red; Exportin-t, panel g, red) and the appearance of unfused, incomplete nuclear membranes (panels e and g, green). However, when exogenous RanQ69L-GTP was added simultaneously with either of these exportins, the resulting nuclear structures excluded TRITC 70 kDa dextran (Crm + Ran, panel f, red; Exportin-t + Ran, panel h, red). The RanGTP was also able to visibly restore the ability to form properly fused nuclear membranes in both Crm1 and Exportin-t reactions, as demonstrated by membrane staining (Crm1, panel f, green; Exportin-t, panel h, green.) Quantification of these results is shown in Figure 2(b), comparing the percent of nuclei which exclude TRITC 70 kDa dextran and have fused membranes (red bars) to the percent of nuclei which do not exclude TRITC 70 kDa dextran and have unfused membranes (black bars) for each condition. For example, Crm1 addition reduces the average % of nuclei with fully enclosed nuclear membranes from 91% (+GST) to 29% (+Crm); however, the addition of RanQ69L-GTP + Crm1 resulted in an average of 77% nuclei excluding TRITC 70 kDa dextran. We conclude that with respect to *in vitro* nuclear reconstitution, Crm1 and Exportin-t can negatively impact the fusion of nuclear membrane precursor vesicles into fully intact double nuclear membranes, leading to an inability to form the correct double membrane barrier that normally separates nucleus from cytoplasm.

### *Crm1 and Exportin-t inhibit spindle assembly in mitotic* Xenopus *egg extracts*

Above, the exportins tested were seen to inhibit nuclear membrane fusion and nuclear pore assembly, events that occur in telophase around the mitotic chromosomes *in vivo*. We asked if exportins could affect an earlier mitotic event: spindle assembly, which begins early in mitosis and culminates at metaphase with the completion of a functional spindle apparatus. In prior studies the import receptors Importin β and Transportin have both been shown to be negative regulators of spindle assembly when added to mitotic *Xenopus* egg extracts [34,35,38,40,50,51,73–77]. *In vivo*, importin beta and RanGTP have also been demonstrated to regulate spindle assembly (see Discussion). Mechanistically, as described in the Introduction, the addition of excess importins has been shown *in vitro* to act by binding and inhibiting spindle assembly factors (SAFs), leading to blocked spindle assembly, and this inhibition can be ameliorated by the simultaneous addition of excess Ran GTP [36].

To ask whether excess Crm1/Exportin1 has an inhibitory effect on spindle assembly in mitotic *Xenopus* egg extracts, spindle assembly reactions were performed by adding demembranated sperm chromatin packets and energy mix to *Xenopus* egg mitotic extracts [50]. Rhodamine-labeled tubulin was also added at the beginning of the reaction to allow for visualization of spindle assembly. Individual mitotic cytosol reactions were then supplemented at t = 0ʹ with: GST alone, or 20 μM GST-Transportin, or different concentrations of recombinant GST-Crm1. At 60 minutes, the reactions were stopped and analyzed for the presence of mitotic spindles (red) around the sperm chromatin packets (blue) (Figure 3).

Spindle assembly was readily observed in the control GST reaction (Figure 3(a), Control, +GST), where a typical robust bipolar spindle assembled around mitotic chromosomes is shown. When tested, addition of low concentrations of Crm1 (1 or 2 μM) had no significant effect on spindle formation (Figure 3(c-d)). Increasing Crm1 concentrations (8 or 10 μM) caused a visible lessening of spindle structure (Figure 3(e-f)). Addition of 15 μM exogenous Crm1 to the mitotic egg extract completely blocked spindle assembly for 80% of the chromatin packets; a typical chromatin packet with an absence of microtubules around the chromatin is shown in Figure 3(g). This inhibition of spindle assembly by Crm1 is similar to that observed following the addition of 15 μM of the importin receptor Transportin, which serves as a negative control (Figure 3(b)) [50,51]. The remaining 20% of chromatin packets at 15 μM Crm1 showed a much reduced level of microtubules arranged in aberrant spindles, or DNA with a few surrounding irregular and disorganized microtubules (data not shown). We conclude that Crm1 addition has an inhibitory effect on spindle assembly *in vitro* that increases with the concentration of Crm1.

Exportin-t was next tested in the *in vitro* spindle assembly assay. Exportin-t (15 μM) addition induced an almost complete lack of any tubulin assembly around the chromosomes (Figure 4(c-d)). Two distinct phenotypes were observed: (1) full inhibition of microtubule assembly into spindles, such that no red microtubules were observed around 52% of the chromatin packets (Figure 4(d)) and (2) mitotic chromatin flanked by very minute aster-like microtubule nucleations (red) for 48% of the DNA packets (Figure 4(c). When RanQ69L-GTP was added simultaneously with Exportin-t (Figure 4(e-h)), it clearly had a partially restorative effect on spindle inhibition, but no normal bipolar spindles were observed. Instead, the main phenotypes observed around the chromatin packets ranged from: chromatin with a very small bipolar spindle adjacent to it but oddly not surrounding the chromatin (Figure 4(e); 34%), red multipolar spindles near the chromatin (Figure 4(f); 16%), and condensed chromatin lacking any microtubule association (Figure 4(h); 5%). Additionally, there were many clusters of asters (Figure 4(g); 45%), similar to those found when RanQ69L-GTP is added by itself to mitotic extract in the absence of DNA as seen in many previous studies. Thus, Exportin-t inhibits mitotic spindle assembly and, although clearly susceptible to partial reversal by RanGTP, in our hands it was never effectively restored by RanGTP to give robust bipolar spindles.

### Crm1 and Exportin-t interact with multiple nucleoporin subcomplexes in pulldowns

Clearly members of the exportin family can inhibit nuclear membrane fusion, nuclear pore formation, and spindle assembly. Although potential targets may abound, nucleoporins offer strong candidate regulatory targets for the inhibition or nuclear pore assembly, as they are known to bind karyopherins during import and many also bind importins in pulldown assays [50–52,78-82].

In the cell a number of nucleoporins are known to be localized not only in the nuclear pore, but also serve as components of the kinetochore at certain cell cycle stages. These Nups have indeed been shown to be required for proper kinetochore assembly and bipolar spindle formation [see, for example, 64, 66]. The nucleoporins that also have kinetochore ‘addresses’ include: the Nup107-160 complex (consisting of Nup107, Nup160, Nup133, Nup96,Nup85, Nup43, Nup37, Sec13, and Seh1), the nucleoporin/kinetochore protein ELYS, the cytoplasmic filament nucleoporin Nup358, Nup214, and a key nucleoporin for import, Nup98 [41,52,78,80–86].

To identify interactions between the exportins used here and nucleoporins in the *Xenopus in vitro* extract, pulldowns were performed using GST-Crm1/Exportin1 beads or GST-Exportin-t beads and *Xenopus* egg interphase extract. Normally, in mature *Xenopus* eggs, the disassembled subunits (derived from oocyte nuclear pores) are stored in large quantity for later in development. These disassembled pore subunits, ~13 subunits in number, were logical targets for testing *importin* regulation of nuclear pore assembly in our past studies with importins [46,47,50,51,87,88]. To ask which, if any, Nup complexes in the extract might be targets of exportin binding, *Xenopus* egg interphase extract was incubated with GST- beads, GST-Crm1 beads, or GST-Exportin-t in the presence or absence of RanQ69L-GTP. Bound proteins were eluted and probed by immunoblotting for the presence of nucleoporins from multiple different nuclear pore subunits, including Nup133 (of the Nup107-160 complex), Nup98 (Gle2), Nup358 (RanGAP1), Nup214 (Nup88), Nup153 (Nup50), and Nup62 (Nup58, 54, 45). (Note that the nucleoporins in parenthesis are Nups which were not probed for here, but are known also to be present in complex with the preceding Nup protein that was probed for.) GST-beads showed no affinity for the nucleoporins tested for (Figure 5, lanes 2–3). However, we found that Crm1 and Exportin-t pulled down all six pore subunits probed for. The blot reveals that Nup133, Nup98, Nup358, Nup214, Nup153, and Nup62 all bound to GST-Crm1 beads and to GST-Exportin-t beads (Figure 5, lanes 4 and 6). Binding indicates that the exportin used must have bound either the nucleoporin probed for, or, another nucleoporin in that subcomplex. When RanQ69L-GTP was included with the exportin-beads during incubation with interphase cytosol, it reduced interactions of the exportins with all the nucleoporins tested (Figure 5, Lanes 5 and 7) and did so significantly with a subset of nucleoporins. We conclude from the pulldowns that nucleoporins, some of which have dual nuclear pore/kinetochore localization, may well be targets of exportin inhibition of nuclear membrane fusion, nuclear pore assembly, and/or spindle assembly. A more complete and detailed analysis of exportin-Nup interactions that are specific to *assembly* events will be required in the future to define the mechanism by which exportins inhibit these key mitotic events.

## Discussion

In this study the question of whether *exportins* regulate or affect major mitotic assembly events was approached. This was done by dissecting the effect of Crm1 and Exportin-t and, to a lesser extent, Exportin-5 on major mitotic assembly events using *in vitro* assays for the separate mitotic events. With respect to nuclear assembly, we found that added Crm1 and Exportin-t did indeed strongly inhibit nuclear pore formation (Figure 1) and also in a separate assay inhibited nuclear membrane fusion (Figure 2). Both types of inhibition could be significantly counteracted by the simultaneous addition of RanGTP. Exportin-5 inhibited nuclear pore assembly and again RanGTP counteracted the inhibition to a large extent (Figure 1). Turning to mitotic spindle assembly, we found that Crm1 and Exportin-t inhibited spindle assembly (Figure 3–4). We are less sure of the reversibility by RanGTP and have resolved to leave this question to the future for those more expert in spindle assembly. Overall, this study shows that the exportins Crm1, Exportin-t, and Exportin-5, when tested, can inhibit major mitotic assembly events and that, in the case of nuclear pore and nuclear membrane assembly, this inhibition is substantially reversed by RanGTP. In search of targets of inhibition, we performed pulldowns and found that Nup358, Nup214, the Nup107-160 complex, and Nup98 pore subunits all showed binding to Crm1- and Exportin-t beads.

For nuclear pore formation, we believe that the release of the nucleoporin subcomplexes from exportins would occur specifically near chromatin where RanGTP is produced (Model, Figure 6). The three exportins examined here in general have very different export cargoes, ranging from Crm1’s diverse array of NES-bearing proteins, to Exportin-t’s tRNA cargo, to the microRNA and specific protein cargos of Exportin-5. Since these cargos are in general very different, we propose below a more parsimonious model where the three exportins act to inhibit nuclear pore assembly by binding to nucleoporins. In this model, the exportins would release the nucleoporins upon interacting with RanGTP, allowing assembly of nuclear pores in forming nuclear membranes near chromatin. In our cell-free assays, the addition of RanGTP promoted this.

For spindle assembly, the addition of the exportins Crm1 and Exportin-t were found to inhibit mitotic spindle assembly (Figures 3–4). Previous studies have revealed that *multiple nucleoporin subcomplexes are required for mitotic spindle assembly*. Those studies demonstrated that nuclear pore proteins have a moonlighting, yet essential role, at the mitotic kinetochore in initiating and stabilizing microtubules as well in other spindle roles [2,52,78,81–85,89]. For example, the Nup107-160 complex has been observed on the spindle microtubules themselves and depletion of the Nup107-160 complex prevents mitotic spindle assembly *in vitro* [81].

What mechanism might explain the inhibition of multiple mitotic assembly events? *(a) Cargo binding*: Crm1 recognizes a huge number of NES-bearing protein cargos, while Exportin-t has much more limited export cargo list, with the most well-known being newly synthesized tRNAs. A model where each exportin studied here inhibits at least one of its own unique *cargos* that happens to be required for each of the three mitotic assembly events would seem unlikely, especially in the case of Exportin-t. Also, a cargo-binding model would seem less likely as RanGTP is in low concentration away from the chromosomes where inhibition would need to occur (see Figure 6). *(b) Ran titration*: The presence of high RanGTP concentrations around the mitotic chromosomes and low RanGTP elsewhere has been shown to be critical for the proper formation of mitotic structures as described in many studies on importins by us and others (see Introduction). It is possible that exportin addition could reduce the available RanGTP needed for Importin β and Transportin to perform their well-known regulatory roles in mitotic structure assembly. However, Ran titration was *not* found to be the model by which the import receptor Transportin regulates mitotic assembly events. Instead, Transportin binds to and inhibits its own set of target proteins needed for mitotic assembly, the most compelling of which are nucleoporin subcomplexes [50]. *(c) Nup binding*: A molecularly parsimonious and broad model, one that would explain the actions of all three exportins tested, would be for the exportins to act by a common mechanism of binding and inhibiting *nucleoporins* essential to the mitotic assembly events. For this model, the exportins would need to bind to the target Nup complexes *in an NES-independent manner*. The exportins would inhibit the Nups in areas distant from the mitotic chromosomes, but release them in the area of high RanGTP around the chromosomes, allowing the Nup complexes to assemble at the forming kinetochore and spindle to promote spindle assembly, and later at the forming nuclear envelope to promote nuclear pore formation. This model conveniently has potential nucleoporin targets that are already known to be needed for both nuclear pore assembly and spindle assembly. As of yet, no specific nucleoporins have been implicated as required for the process of nuclear membrane assembly.

This Nup-binding model is strengthened by our GST-exportin-bead pulldowns from *Xenopus* interphase extracts, which contain the disassembled subunits of nuclear pores. When the pulldowns were performed, Crm1- and Exportin-t beads were found to associate with multiple nucleoporin subcomplexes and the binding was diminished for most, but not all, of the nucleoporin subcomplexes by the inclusion of RanGTP. Each exportin brought down a similar set of multiple Nups, consistent with a common mechanism. Each Nup subunit that bound has been previously implicated in spindle assembly or function, and clearly is also important to nuclear pore assembly. We believe the most cohesive model would involve the exportins – via a karyopherin domain not involved in cargo binding – interacting with the Nups with this binding being reversed by RanGTP near chromatin, just as we see in the pulldowns.

Indeed, a significant body of *in vivo* evidence for karyopherin regulation of Nups in mitosis abounds. This includes the following: (1) Injection of Importin β fragments into mammalian cells leads to severe defects in spindle microtubule and chromosome morphologies [38]. (2) Importin β overexpression in cells causes microtubule fragmentation; this can be rescued by the co-expression of TPX2, a spindle assembly factor shown to be inhibited by Importin β [90, 74]. (3) In human cells, Nup358 (RanBP2) with RanGAP1 have been shown to colocalize with Crm1 as a complex on kinetochores in metaphase [73,76,89]. Indeed, Crm1 is *required* for localization of Nup358/RanGAP1 to the kinetochore. Most importantly, Crm1, Nup358 and RanGAP1 have all been shown to be necessary to *stabilize* the kinetochore-microtubule interaction in mitosis [89]. (4) A parallel study showed that Crm1 is phosphorylated by mitotic kinase CDK1 and similarly saw Crm1localize to the mitotic spindle and kinetochores during metaphase [91]. (5) Crm1 has been shown to play a role in centrosome duplication, targeting nucleophosmin to the centrosome in an NES-dependent manner [92]. (6) Importin β/Nup358/RanGAP1 was seen on spindle microtubules early in mitosis and appears to transfer Nup358/RanGAP1 to Crm1 at the kinetochores later in mitosis [73]. This in vivo evidence has influenced our mechanistic considerations to favor a ‘GPS’-like model where at mitosis the exportins inhibit Nup subunits needed for mitotic assembly events and free them only where there is high RanGTP.

In sum, the *in vitro* evidence presented here provides new insight into novel regulatory mechanisms the cell could employ to ensure correct spatial arrangement of major mitotic structures for a faithful segregation of mitotic chromosomes, followed then by proper nuclear reassembly. This would greatly expand the current known roles of RanGTP and karyopherin regulation of major mitotic assembly events to include the exportins.
